# Three-Dimensional Cellular Automata Simulation of the Austenitizing Process in GCr15 Bearing Steel

**DOI:** 10.3390/ma12183022

**Published:** 2019-09-18

**Authors:** Fuyong Su, Wenli Liu, Zhi Wen

**Affiliations:** School of Energy and Environmental Engineering, University of Science and Technology Beijing, Beijing 100083, China; liuwenliwyyx@163.com (W.L.); wenzhi@me.ustb.edu.cn (Z.W.)

**Keywords:** three-dimensional cellular automaton model, austenitizing process, interfacial movement velocity

## Abstract

On the basis of the two-dimensional cellular automaton model, a three-dimensional cellular automaton model of austenitizing process was established. By considering the orientation of pearlite layer and the direction of austenite grain growth, the velocity of the interface was calculated during the austenitizing process. The austenitizing process of GCr15 steel was simulated, and the anisotropy of grain growth rate during austenitization was demonstrated by simulation results. By comparing the simulation results with the experimental data, it was found that the calculated results of the three-dimensional cellular automaton model established in this paper were in good agreement with the experimental results. By using this model, the three-dimensional austenitizing process of GCr15 steel at different temperatures and under different processing times can be analyzed, and the degree of austenitization can be predicted.

## 1. Introduction

The structure of the bearing steel continuous casting billet at normal atmospheric temperature is mainly composed of lamellar pearlite structure and carbide. Pearlite is a mixed structure formed by the interleaving of lamellar ferrite and cementite. When the steel temperature exceeds the transition temperature, the steel can spontaneously be an austenitizing transformation process. It is generally believed that the austenitizing process is a transformation process of nucleation growth. The nucleation rate of the nuclei and the growth rate of the grains together determine the rate of the austenitizing transformation process.

Speiche et al. [1] pointed out that austenite can be nucleated at the interface between cementite and ferrite. Roosz et al. [2] believed that austenite can nucleate at three interfaces of cementites and ferrites, which are the internal interface of pearlite group, the interface on the edge of pearlite, and the interface on the corner of pearlite. The relationship was obtained between the nucleation rate of austenite and the morphology of pearlite by experiment. Shtansky et al. [3] observed the austenite nucleation inside and at the boundary of the pearlite by using transmission electron microscopy. Another view is that austenite nucleation is mainly at the boundary of pearlite clusters [4]. Li et al. [5] further pointed out that austenite mainly nucleated at the interface of high-angle pearlite. Combining the two viewpoints, it can be considered that austenite mainly nucleates at the boundary of the pearlite group, and also has a certain nucleation inside the pearlite group.

For the austenitizing process, in addition to experimental observation of the metallographic phase of the sample, many scholars have also carried out numerical simulation studies on the transformation process of pearlite to austenite. Akbay et al. [6] established a simple model for the transformation of lamellar ferrite and cementite into austenite, and obtained analytical solutions and numerical results under steady state conditions. Kong et al. [7] proposed a fixed integral formula of austenite volume fraction during uniform heating on the basis of experiments, and obtained an approximate analytical solution. Caballero et al. [8,9] obtained the definite integral formula of austenitization in isothermal transformation process and uniform heating process based on the semi-empirical nucleation rate and growth rate formula proposed by Roosz et al. [2], and the results had a high consistency with the experimental results. Some scholars had obtained a simplified model of the moving velocity of austenite and pearlite interface by analyzing the diffusion process, and combined it with the Avrami model to obtain the definite integral expression of austenite volume fraction [10,11].

Commonly used methods for numerical simulation include the diffusion field model, the cellular automaton, and the phase-field. Among them, the cellular automata method has attracted much attention because of its advantages in dynamic system simulation. In simulation, by using the cellular automaton, many scholars regard the transformation of pearlite to austenite as a result of random transitions of the cells. The driving force of the transition is the sum of the energy barrier and the difference of free energy between the two states. Therefore, the difficulty of this method lies in the determination of the driving force [12,13,14]. Su et al. [15] established the cellular automaton model based on the semi-empirical austenite nucleation and growth formula proposed by Roosz et al. [2]. This cellular automaton model can obtain the micro-organization of the austenitizing transformation process. On the basis of the above two-dimensional cellular automaton model, a three-dimensional model of the austenitizing transformation process of GCr15 steel was established by using the cellular automata method, and the transformation process of pearlite to austenite was simulated.

## 2. Three-Dimensional Cellular Automaton Model

The cellular automaton model is a mathematical model that is time-discrete and spatially discrete. Each discrete point is assigned a state parameter whose possible states are also discrete and finite. The cellular automaton model follows the rules of local evolution. In the evolution process of each time step, the state of a certain point is determined by the state of the points around it, defined as neighbors, and is independent of other points. With the cellular automaton model, many complex continuous changes can be discretized into simple local evolution processes to achieve the reproduction of some complex phenomena.

A cellular automaton model consists of cells, state of the cell, cell space, neighbor’s types, and evolution rules.

**Figure 1:**
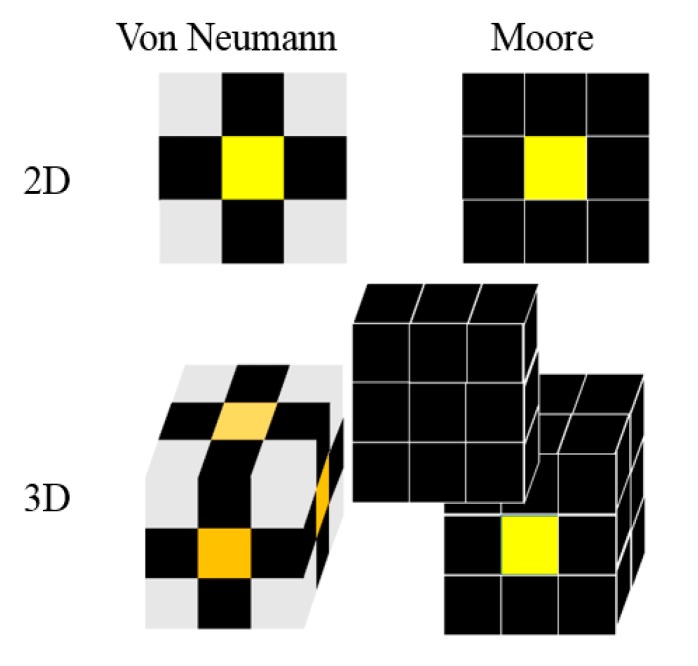
Two kinds of neighbor’s type.

(1) The cell. The cell is the basic unit of the cellular automaton model, and is the carrier of various state parameters and the executor of the evolution rules. In the simulation of the evolution of the material, the cell is the unit of material that is discretized. For a two-dimensional model, the shape of the cell is mainly square, regular hexagon, and equilateral triangle. For a three-dimensional model, the shape of the cell is mainly truncated octahedron, cube, and sphere.

(2) The state of the cell. The state of a cell includes all states of a finite number of discrete material. In principle, one cell can only have one state, but multiple states can exist simultaneously in the actual application process.

(3) The cell space. The space is a collection of the cells. The set of multiple cells can be seen as a cell space.

(4) The neighbor’s type. For a specific lattice point, the lattice points of the local area that are involved in the evolution rule are called the neighbor lattice point. The distance between one lattice point and the specific lattice point is generally used to determine whether the lattice point is the neighbor. For a space composed of square or cube cells, there are three main types of cells, namely Von Neumann type neighbors and Moore type neighbors, as shown in Figure 1.

(5) The evolution rules. The cell and cell space are the basic unit of the model. To realize the dynamic evolution process of the model, it is necessary to add the evolution rule. The evolution rule is built according to a specific physical process. In the evolution rule, only the state of the specific lattice point and its neighbor’s type can be considered to determine the state of the specific lattice point at the next moment.

The boundary conditions of the cellular automaton model mainly include fixed boundary conditions, symmetric boundary conditions, and periodic boundary conditions. The periodic boundary condition was used in the calculations in this paper. During the calculation process, all lattice points were updated synchronously.

## 3. Three-Dimensional Cellular Automata Simulation of the Austenitizing Process

The austenitizing process can be decomposed into three processes: nucleation, austenite nucleus growth, and austenite grain collision. The nucleation process occurs on the pearlite matrix and is related to the local morphology of the pearlite matrix and the driving force of the pearlite phase transition. In general, the trigeminal intersection and interface of the pearlite mass are easier to nucleate, and the nuclei may also be formed between the two pearlite layers. After the nucleation process is completed, the newly formed austenite nuclei form a new phase interface with the pearlite matrix. The phase interface is generally propelled to the pearlite matrix under the action of diffusion driving force, so that the pearlite transforms into austenite. The moving velocity of the interface between the austenite and the pearlite is related to the solute diffusion coefficient in the material and the thickness of the pearlite layer. As the austenite nucleus continues to grow, when the two austenite grains meet at a certain point, the interface between the pearlite and the austenite transforms into an austenite grain boundary, that is, the austenite grain collision. The two collided austenite grains will continue to move caused by the grain boundary curvature, that is, the austenite grain growth.

### 3.1. Mathematical Description of the Austenitizing Process

**Figure 2:**
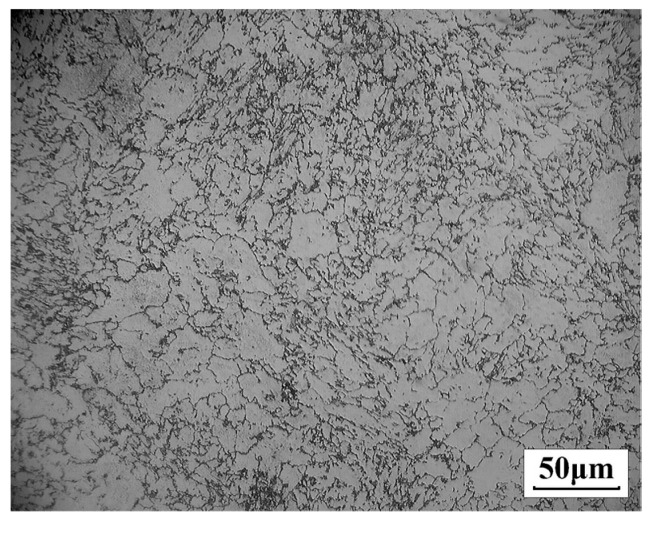
Metallographic diagram after austenitization.

Speiche et al. believe that the austenite nucleation process is instantaneous, that is, the nucleation position has been exhausted in the initial stage of austenitizing transformation. However, Roosz et al. believe that the austenite nucleation process is continuous, that is, the nucleation rate remains constant during the austenitizing transformation process. In the study by Speiche et al., the C content of the steel was 0.96%, and in the study by Roosz et al., the C content of the steel was 0.78%. Studies by Dernfeld [16] have confirmed that this difference in nucleation is mainly due to differences in C content. The composition of GCr15 steel studied in this paper is shown in Table 1. Its C content was about 1%, which is closer to Speiche’s research. It can be considered that the nucleation process was instantaneous. Therefore, in this paper, the number of austenite grains in the sample which was rapidly cooled after complete austenitization was used, instead of the number of austenite nuclei at the beginning of the transformation, thereby obtaining the number of austenite nuclei per unit volume. As shown in Figure 2, after more than 1000 grains, the instantaneous nucleation density (N/V) of austenite was 1.687 × 1015 m^−3^. N is the number of austenite nuclei and V is the volume.

The interfacial moving velocity of austenite in different directions determines the austenitizing process of GCr15 steel. The research on the interface velocity of austenite growth in pearlite mainly includes numerical simulation based on solute diffusion and the nucleation and growth model that was proposed by Roósz et al. [2] on the basis of experimental research. Gaude-Fugarolas et al. [17] proposed that the austenitizing process is controlled by C diffusion, and the interface velocity can be calculated as follows:

(1) $$\bar{v}=\frac{1}{r_{f}-r_{0}}(\ln r_{f}+\ln r_{0})D(\frac{c^{\gamma \theta }-c^{\gamma \alpha }}{c^{\gamma \alpha }-c^{\alpha \gamma }}),$$

where $\bar{v}$ is the average velocity of the austenite interface, in meters per second; *r_f_* and *r*_0_ are the farthest and closest distances of diffusion, respectively, in meters; *r_f_* is half of the thickness of the pearlite layer; *r*_0_ is a few lattice thicknesses, about 10^−8^ m; *D* is the diffusion coefficient of the main element’s diffusion in austenite, in meters squared per second; *c^γθ^* is the molar concentration of solute in austenite at the interface between austenite and cementite; *c^γα^* is the molar concentration of solute in austenite at the interface between austenite and ferrite; and *c^αγ^* is the molar concentration of solute in ferrite at the interface between ferrite and austenite.

**Figure 3:**
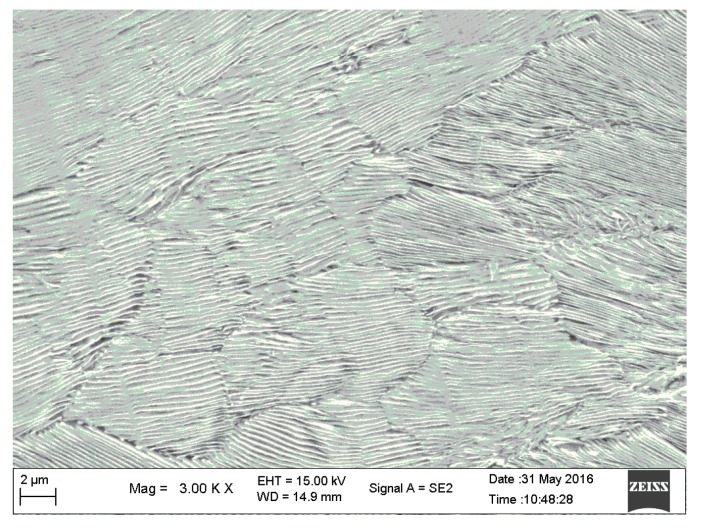
SEM image of pearlite layer structure.

For austenitizing process and cementite dissolution processes in high carbon low alloy steels, Hillert [18] proposed two models. In the case of low superheat, the pearlite dissolution process depended on the diffusion of alloying elements; when the temperature is above a certain critical temperature, the pearlite dissolution process does not depended on the diffusion of alloying elements, and the austenitizing process depends on diffusion of carbon elements. Because of the difficulty in calculating the transition temperature, it is difficult to determine the solute elements, and thus it is difficult to implement the model in Equation (1). Therefore, this paper uses the semi-empirical model proposed by Roósz:

(2) $$v=E\frac{1}{\sigma_{0}^{2}}\exp\left(-\frac{Q}{k\Delta T}\right),$$

where *E* is the empirical constant and *Q* is the austenite growth process activation energy. According to the experimental results [19], the empirical constant and activation energy are 3.406 × 10^−19^ m^3^/s and 6.995 × 10^−22^ J/atom, respectively. k is the Boltzmann constant, ΔT is the degree of superheat, and *σ*_0_ is the pearlite layer spacing. As shown in Figure 3, the measured value of *σ*_0_ was 0.227 μm.

**Figure 4:**
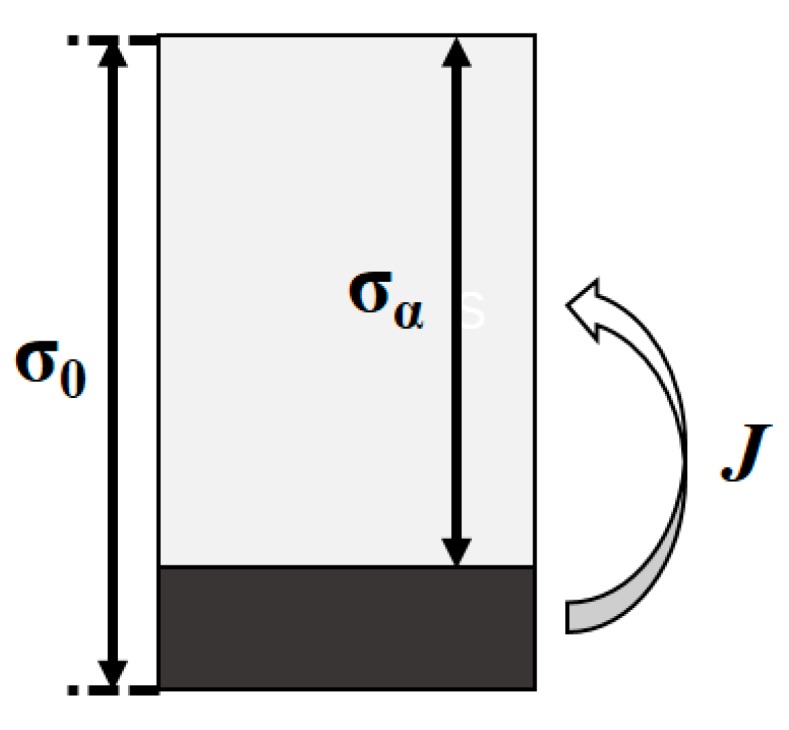
Diffusion near the interface.

The above is the austenite boundary perpendicular to the direction of the pearlite layer, that is, the austenite growth rate was calculated when the austenite interface growth direction was parallel to the pearlite layer. According to the study of reference [20], it can be seen that the direction in which austenite grains move in the pearlite had a significant effect on the austenite interface moving velocity. The austenite growth process was solute atoms diffusing from cementite through austenite to ferrite. Therefore, the following flow conservation relationship should be satisfied at the austenite and ferrite interface:

(3) $$v(c^{\gamma \alpha }-c^{\alpha \gamma })=J/\sigma_{\alpha },$$

where *J* is the solute flow rate through austenite, in mol·s^−1^·m^−2^. *σ_α_* is the thickness of the ferrite layer, as shown in Figure 4, in meters. The diffusion distance from cementite to ferrite is 1/2 of the thickness of the layer, so the flow rate *J* can be approximated by the following formula:

(4) $$J=\frac{2(c^{\gamma \theta }-c^{\gamma \alpha })}{\sigma_{0}}.$$

The two distance parameters (*σ*_0_ and *σ_α_*) in Equations (3) and (4) are proportional to the spacing of the layers, and it can be inferred that the austenite interface moving velocity is inversely proportional to the square of the layer thickness. This result is consistent with the form in the semi-empirical model Equation (2).

**Figure 5:**
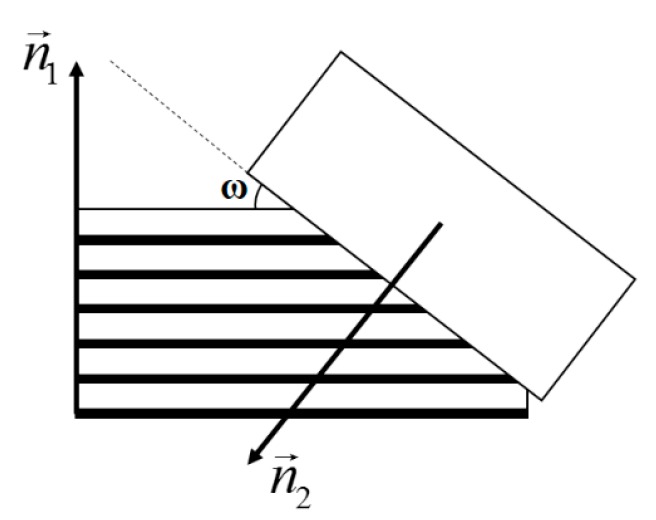
Schematic diagram of the movement direction of the austenite interface.

As shown in Figure 5, when the direction of austenite growth is at an angle to the direction of the pearlite layer, the distance parameters in Equations (3) and (4) are multiplied by 1/sin*ω*. Considering this situation, the moving velocity in Equation (2) will be calculated as follows:

(5) $$v_{1}=E\frac{1}{\sigma_{0}^{2}}\exp\left(-\frac{Q}{k\Delta T}\right)(1-\cos^{2}\omega ),$$

(6) $$\cos\omega =\frac{\left|{\vec{n}}_{1}\cdot {\vec{n}}_{2}\right|}{\left|{\vec{n}}_{1}\right|\left|{\vec{n}}_{2}\right|},$$

where ${\vec{n}}_{1}$ is the normal vector of the pearlite layer and ${\vec{n}}_{2}$ is the moving direction vector of the austenite grain boundary. In the calculation process, the ${\vec{n}}_{1}$ is the normal vector which is assigned randomly by the program in the initial tissue formation process of pearlite, and the same pearlite group has the same value. The ${\vec{n}}_{2}$ is related to the relative position of the austenite point and the pearlite point. When the position of the interface pearlite grid is determined, a vector can be determined relative to a certain austenite neighbor.

### 3.2. Simulation Results and Analysis

A three-dimensional cellular automaton model was established in this paper. The shape of the cell was a cube, and the neighbor type was a Moore-type neighbor. There were two states of the cell, one state was pearlite, and the other was austenite. For each pearlite cell, a laminar normal direction vector was assigned, and adjacent cells having the same normal vector formed a pearlite cluster. For each newly formed austenite nucleus, an austenite orientation was imparted, austenite grains were formed by austenite nucleation, and new austenite was formed by austenite nucleus growth. The orientation of the newly formed austenite cell was the same as the orientation of the austenite nuclei.

**Figure 6:**
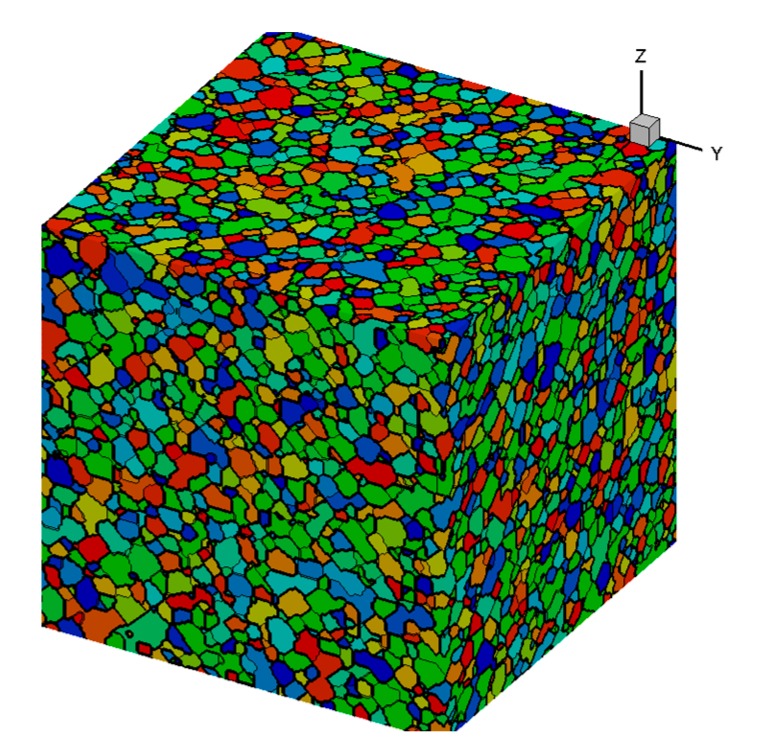
Initial organization of pearlite.

In this paper, the C#.net was used to program the three-dimensional cellular automaton model of the austenitizing process of bearing steel, and the simulation was carried out using the self-compiled program. The initial organization of the pearlite was formed by the Monte-Carlo method when the calculation was started, as shown in Figure 6. The size of the pearlite in the initial structure was the same as the pearlite measured in the actual initial structure [19]. According to the conclusions in reference [5], it was assumed that the austenite nuclei were formed at the trigeminal boundary of the pearlite cluster. The austenite nucleation process was instantaneous, and no new austenite nucleation was formed during the growth of austenite grains.

In this paper, there were three kinds of distance between the central cell and the neighbor cell. The first one was the cell being coplanar with the central cell; the distance was a cell side length *a*. The second was the cell being co-edge with the center cell; the distance was $\sqrt{2}a$. The third was the cell being co-apex with the central cell, and the distance was $\sqrt{3}a$. For a pearlite cell, when a neighbor cell was austenite, the probability that the center cell translated to austenite under the action of the neighbor cell was determined by the following equation:

(7) $$p=v_{1}\Delta t/L,$$

where *L* is the distance between the neighbor and the central cell, in meters and Δ*t* is the calculated time step, in seconds. The austenite interface moving velocity *v*_1_ is determined by Equation (5), and the moving direction of the austenite grain boundary is related to the direction of the pearlite layer. The normal vector ${\vec{n}}_{1}$ of the pearlite layer is randomly assigned in the initial tissue. The direction of austenite movement ${\vec{n}}_{2}$ is a vector pointing from the center of the neighboring austenite cell to the center cell, which can be calculated from the coordinates of the cell. The transformation of the central cell is the result of the action of all austenitic cells in its neighbors, so the total transition probability of the central cell is

(8) $$P=\sum p_{i},$$

where *p_i_* is the transition probability of the action of the austenite cell *i* on the central cell in the neighbor cell.

**Figure 7:**
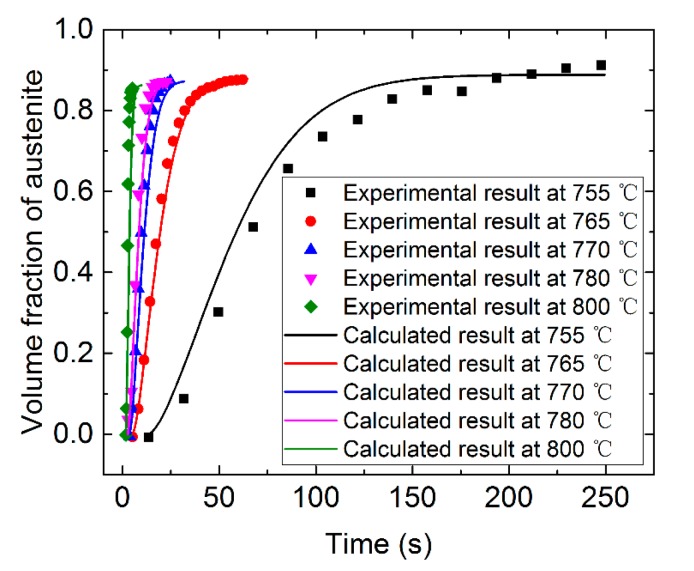
Calculation and experimental results of isothermal austenitizing process.

Using this model, the austenitizing process was calculated under isothermal conditions, and the calculated temperatures were 755 °C, 765 °C, 770 °C, 780 °C, and 800 °C. The calculation results are shown in Figure 7. It can be seen from the figure that the calculation results are in good agreement with the experimental results [19]. The maximum relative error at 765 °C, 770 °C, 780 °C, and 800 °C was less than 4%, and the maximum relative error at 755 °C was less than 8%. This indicated that the cellular automaton model proposed in this paper can better simulate the transformation process of pearlite to austenite. It can also be seen from the figure that with the initial organization of the pearlite of this paper, the time when 80% of the pearlite transformation to austenite was at about 5 s, 10 s, 15 s, 27 s, and 100 s at 800 °C, 780 °C, 770 °C, 765 °C, and 755 °C, respectively. Increasing the temperature can speed up the conversion of pearlite to austenite. When the temperature was increased from 755 °C to 765 °C, the conversion speed was increased by about four times. When the temperature was increased to 770 °C, the conversion speed was increased by about seven times. When the temperature was increased to 780 °C, the conversion speed was increased by about 10 times. When the temperature was increased to 800 °C, the conversion was almost instantaneous.

**Figure 8:**
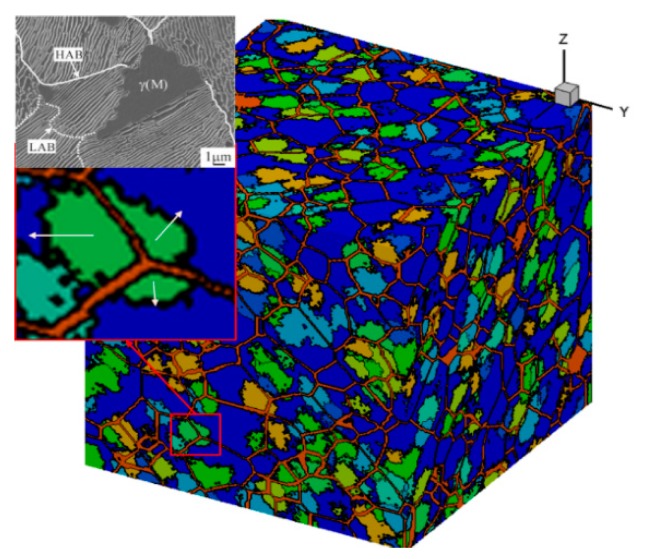
Grain morphology after 50% transformation of austenitization.

While accurately simulating the austenitizing process, the model of this paper can also clearly demonstrate the anisotropy caused by the direction of the pearlite layer during austenite grain growth. As shown in Figure 8, the orange line in the figure is the boundary of the original pearlite mass, and the position of the austenite nucleus is at the trigeminal junction of the pearlite mass. It can be seen from the figure that the velocity of austenite grains growing in all directions was not the same, similar to the phenomenon observed in the experimental diagram. The cellular automaton model in this paper reproduced the anisotropy of austenite growth. Because of the different conditions of the growth process, the grains with better growth conditions obtained a relatively large volume, while the grains with poor growth conditions had a small volume.

## 4. Conclusions

In this paper, a three-dimensional cellular automaton model for the transformation of bearing steel pearlite to austenite was established. In the austenitizing process, because of the angle between the orientation of the pearlite and the growth direction of austenite, the austenite had different growth velocity in different directions. In this paper, the austenitizing process of pearlite was predicted by the three-dimensional cellular automaton model, and the expression of interfacial movement velocity of pearlite layer orientation and austenite grain growth direction was considered comprehensively. The anisotropy of grain growth in the pearlite was analyzed. The calculation results of the three-dimensional cellular automaton model in isothermal condition were in good agreement with the experimental results; the maximum relative error between calculation and experimental results at 765 °C, 770 °C, 780 °C, and 800 °C was less than 4%, and the maximum relative error at 755 °C was less than 8%. Increasing the temperature can speed up the conversion of pearlite to austenite. When the temperature was increased from 755 °C to 765 °C, 770 °C and 780 °C, the conversion speed was increased by about 4 times, 7 times, 10 times, respectively; when the temperature was increased to 800 °C, the conversion was almost instantaneous.

## Figures and Tables

**Table 1:** Composition of GCr15 steel.

Component	C	Si	P	S	Fe
Mass fraction (%)	1	0.25	0.25	0.25	Bal.
component	Cr	Mn	Mo	Ni	Cu
Mass fraction (%)	1.5	0.35	0.1	0.30	0.25
